# The Interrelationship Between Microbiota and Peptides During Ripening as a Driver for Parmigiano Reggiano Cheese Quality

**DOI:** 10.3389/fmicb.2020.581658

**Published:** 2020-10-02

**Authors:** Benedetta Bottari, Alessia Levante, Elena Bancalari, Stefano Sforza, Chiara Bottesini, Barbara Prandi, Francesca De Filippis, Danilo Ercolini, Marco Nocetti, Monica Gatti

**Affiliations:** ^1^Department of Food and Drug, University of Parma, Parma, Italy; ^2^Department of Agricultural Sciences, University of Naples Federico II, Naples, Italy; ^3^Task Force on Microbiome Studies, University of Naples Federico II, Naples, Italy; ^4^Consorzio del Formaggio Parmigiano-Reggiano, Reggio Emilia, Italy

**Keywords:** cheese microbiota, cheese peptides, raw milk cheese, cheese ripening, Parmigiano Reggiano

## Abstract

Cheese microbiota contribute significantly to the final characteristics of cheeses due to the growth and interaction between cheese microorganisms during processing and ripening. For raw milk cheeses, such as Parmigiano Reggiano (PR), the microbiota derive from the raw milk itself, the dairy environment, and the starter. The process of cheese making and time of ripening shape this complex ecosystem through the selection of different species and biotypes that will drive the quality of the final product by performing functions of their metabolism such as proteolysis. The diversity in the final peptide and amino acid composition of the cheese is thus mostly linked to the diversity of this microbiota. The purpose of this study was to get more insight into the factors affecting PR cheese diversity and, more specifically, to evaluate whether the composition of the bacterial community of cheeses along with the specific peptide composition are more affected by the ripening times or by the cheese making process. To this end, the microbiota and the peptide fractions of 69 cheese samples (from curd to cheese ripened 24 months) were analyzed during 6 complete PR production cycles, which were performed in six different dairies located in the PR production area. The relation among microbial dynamics, peptide evolution, and ripening times were investigated in this unique and tightly controlled production and sampling set up. The study of microbial and peptide moieties in products from different dairies – from curd to at least 12 months, the earliest time from which the cheese can be sold, and up to a maximum of 24 months of ripening – highlighted the presence of differences between samples coming from different dairies, probably due to small differences in the cheese making process. Besides these differences, however, ripening time had by far the greatest impact on microbial dynamics and, consequently, on peptide composition.

## Introduction

Cheese microbiota is a complex ecosystem that can originate from raw milk, acidifying starters and adjunct cultures, and adventitious microorganisms that may come from equipment and the cheese making plant environment. The cheese microbiota is then shaped by the cheese making steps and ripening, which cause a selective pressure on microorganisms. Given this framework, other authors have underlined the importance of studying cheese microbial dynamics to better understand their effects on both quality and safety (Kamimura et al., 2019). In particular, the succession of different microbial groups and their interaction during cheese making and ripening is fundamental for the development of the unique sensory characteristics of each cheese variety (Gobbetti et al., 2018; Afshari et al., 2020). In raw milk, long ripened hard cheeses, such as Parmigiano Reggiano (PR), lactic acid bacteria (LAB) play a major role both as starters (SLAB) in curd acidification and non-starter (NSLAB) during cheese-ripening (Gatti et al., 2014; Carafa et al., 2019; Martini et al., 2020). In addition to their fermentation performances, LAB have attracted remarkable interest in the last decades for their potential health benefits through the consumption of fermented foods (De Filippis et al., 2020), and many LAB species have recently been linked to the gut microbiota in a genome-wide analysis (Pasolli et al., 2020).

Parmigiano Reggiano (PR) is an internationally appreciated, protected designation of origin (PDO) Italian cheese, artisanally produced in multiple dairies in the PDO area. It is made under strict production regulation, but small differences in operational conditions in each dairy may occur. These result in different cheese microbiota compositions, metabolites and their inter-relationships that underpin specific cheese quality attributes (Gatti et al., 2014). During cheese ripening, a complex chain of events occurs that entails a set of biochemical reactions, and proteolysis is one of the most important. Proteolysis is initiated by the starter, continued by non-starters, and completed and tailored by the proteolytic enzymes released by the bacterial community. These events lead to changes in the specific peptide and amino acid composition that constantly evolves during the aging period. Sforza et al. (2012) correlated these trends in peptides’ evolution to the enzymatic activities, thus allowing for the discrimination of cheeses according to their aging times. That said, the aim of this work was to get a deeper insight into the factors affecting PR diversity by evaluating whether the composition of the bacterial community and the specific peptide composition are more affected by the ripening times or by the cheese making process. To this end, the microbiota and the peptide fractions of 69 cheese samples (from curd to cheese ripened 24 months) were analyzed during 6 complete PR production cycles, which were performed in six different dairies. Bacterial dynamics were studied taking into account both total DNA extracted from cheeses and DNA from entire or lysed cells, thus permitting a better understanding of proteolysis and peptide evolution during PR cheese ripening in the context of a unique and tightly controlled production and sampling set up.

## Materials and Methods

### Cheese Sampling

**FIGURE 1:**
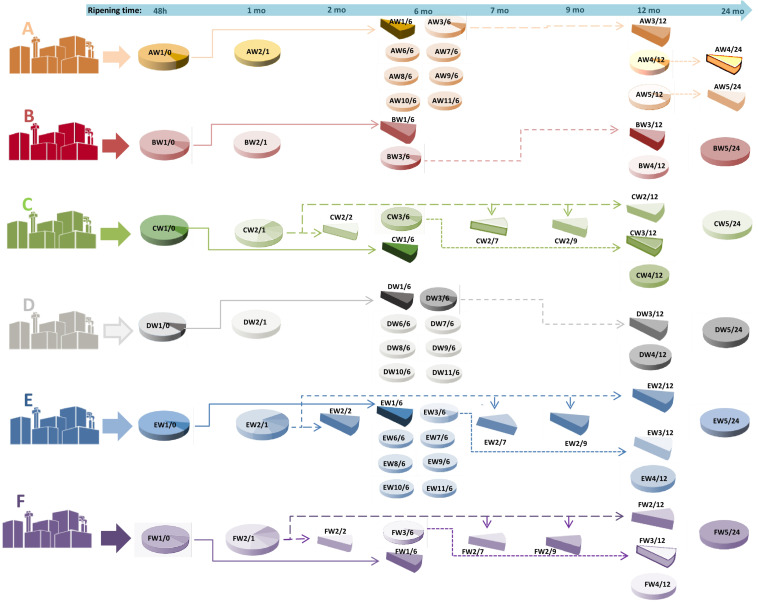
A graphical representation of the sampling scheme. For each dairy **(A–F)**, samples were taken from the same cheese making lot (same wheel, W) at different ripening stages, and from different cheese making lots (different wheels) at the same ripening stage. As an example, AW1/0 corresponds to dairy A, wheel 1, months of ripening 0, that is to say, the curd after 48 h from cheese-making; AW1/6 is the same wheel sampled after 6 months. Samples from the same cheese making lot are connected by arrows. The time of ripening is also indicated at the top of the picture by an arrow.

Cheese samples were obtained from the “Consorzio del Parmigiano-Reggiano” (Reggio Emilia, Italy). Six dairies (designated as A–F) located in the PR PDO production area were considered for this study. For each dairy, samples were taken from the acidified curd (48 h), after brining (1 month of aging) and after 6, 12, and 24 months. For dairy C, E, and F, samples were also taken at 2, 7, and 9 months. To evaluate the microbial and peptide dynamics over time, samples were taken at different ripening times from the same original wheel. Moreover, samples were taken for each dairy from different wheels with the same ripening times (Figure 1 and Table 1). A total of 69 samples were collected: 48 h (6 curd samples), 1-month-old (6 cheese samples), 2-month-old (3 cheese samples), 6-month-old (27 cheese samples), 7-month-old (3 cheese samples), 9-month-old (3 cheese samples), 12-month-old (15 cheese samples) and 24-month-old (six cheese samples). For each dairy (A–F), samples were identified with the letter W followed by a number, indicating the sampled wheel, and a slash followed by a second number, indicating the stage of ripening (e.g.,: AW1/0 corresponds to dairy A, wheel 1, months of ripening 0, that is to say, the curd 48 h after cheese-making).

**TABLE 1:** Sampling scheme.

Ripening stages								

Dairy	48 h (acidified curd)	1 month (end of brining)	2 month	6 month	7 month	9 month	12 month	24 month
A	W1/0	W2/1	–	W1/6, W3/6, W6/6, W7/6, W8/6, W9/6, W10/6, W11/6	–	–	W3/12, W4/12, W5/12	W4/24, W5/24
B	W1/0	W2/1	–	W1/6, W3/6	–	–	W3/12, W4/12	W5/24
C	W1/0	W2/1	W2/2	W1/6, W3/6,	W2/7	W2/9	W2/12, W3/12, W4/12	W5/24
D	W1/0	W2/1	–	W1/6, W3/6, W6/6, W7/6, W8/6, W9/6, W10/6, W11/6	–	–	W3/12, W4/12	W5/24
E	W1/0	W2/1	W2/2	W1/6, W3/6, W6/6, W7/6, W8/6, W9/6, W10/6, W11/7	W2/7	W2/9	W2/12, W3/12, W4/12	W5/24
F	W1/0	W2/1	W2/2	W1/6, W3/6,	W2/7	W2/9	W2/12, W3/12, W4/12	W5/24

*Samples taken from the same wheel have the same W number. The number after each slash indicates the month of ripening. Dairies are written with capital letters (A–F).*

Cheeses were produced according to EU Regulation of PDO established by article 11 of regulation (EU) No. 1151/2012 (European Commission, 2012). According to this regulation, the cheese making procedure is the same for all the dairies with small variations due to operational conditions that may occur among the dairies, variations which were not considered as a relevant variable for the present study (Gatti et al., 2014). Milk from the previous day, partially skimmed by spontaneous floating, was mixed with fresh whole milk in copper vats. Calf rennet and natural whey starter were added. The curd was broken and heated at 55°C for about 50 min. Curd was then extracted and molded for 48 h, before brining. After 1 month, the wheels were extracted, washed, and ripened. Samples were obtained by coring, thus obtaining a transverse section for each wheel. Each entire section was grated and mixed before the analysis in order to have a sample representative of the whole wheel. Two separate aliquots were then prepared: one was immediately analyzed and one was kept at −20°C for the subsequent DNA and cheese water soluble extraction.

### Microbial Counts

Ten grams of each grated cheese sample were suspended in 90 ml of 20 g/L trisodium citrate (pH 7.5) (Sigma–Aldrich, St. Louis, United States) and homogenized for 2 min in a blender (Seward, London, United Kingdom). Decimal dilutions of milk and homogenates were made in quarter-strength Ringer solution (Oxoid, Basingstoke, United Kingdom) and spread-plated in triplicate on the appropriate medium described as follows. Cheese agar medium (CA) (Neviani et al., 2009) was used for the enumeration of cultivable NSLAB population, incubating plates at 37°C, while SLAB were counted on De Man, Rogosa and Sharpe (MRS) agar (Oxoid, Basingstoke, United Kingdom), incubating plates at 42°C. All the plates were incubated for 2 days under anaerobic conditions.

### Culture-Independent Viable Counts

The number of viable cells was obtained by using the LIVE/DEAD^®^ Baclight^TM^ Bacterial Viability kit (Molecular Probes, Oregon, United States) and fluorescence microscopy (Gatti et al., 2006). The grated cheese homogenates in trisodium citrate (15 ml) were centrifuged at 10,000 rpm for 10 min at 4°C. The obtained pellets were washed twice in 15 ml 20 g/L trisodium citrate (pH 7.5) (Sigma–Aldrich, St. Louis, United States), then resuspended in 15 ml sterile water and 10-fold diluted. Subsequently, 1 ml of each sample was used for viability counts according to the manufacturer’s instructions. Samples stained with LIVE/DEAD^®^ Baclight^TM^ were then filtered onto black polycarbonate filters (0.2 μm pore size) (Millipore Corp., Billerica, MA, United States), visualized by an epifluorescence microscope (Nikon 80i, Tokyo, Japan) and counted as described by Bottari et al. (2010). Three separate counts were performed for each sample. Results were expressed as viable cells and total cells, resulting from the sum of viable and non-viable cells (cells/mL or cells/g).

### DNA Extraction

The total DNA for high throughput 16S rRNA sequencing was extracted from 69 samples using DNeasy Blood and Tissue Kit (Qiagen, Hilden, Germany), as described in Bertani et al. (2020). Bacterial genomic DNA for LH-PCR (length heterogeneity polymerase chain reaction) analysis was extracted using the silica column method with the General Rapid Easy Extraction System (GREES) DNA kit (InCura S.r.l., Cremona, Italy), according to the manufacturer’s instructions. Two, 6, 9, 12, and 24-month-old cheeses were treated to extract DNA both from whole and lysed cells (Gatti et al., 2008; Santarelli et al., 2013).

### High Throughput 16S rRNA Sequencing and Bioinformatic Data Analysis

Microbial diversity was studied through the sequencing of the amplified V3-V4 region of the 16S rRNA gene by using primers S-D-Bact-0341-b- S-17: 5′-CCTACGGGNGGCWGCAG-3′ and S-D-Bact-0785-a-A-21: 5′- GACTACHVGGGTATCTAATCC-3′ amplifying a fragment of 464 bp (Klindworth et al., 2013). Library preparation and sequencing was carried out as previously described (Berni Canani et al., 2017). The amplicons were purified using AMPure Beads XT (Beckman Coulter), quantified using a fluorimeter and combined in an equimolar pool, which was sequenced on an Illumina MiSeq platform, leading to 2x250 bp reads.

After demultiplexing, paired-end reads were joined by FLASh (Magoč and Salzberg, 2011) and a quality filtering was carried out by PRINSEQ (Schmieder and Edwards, 2011). Reads were trimmed at the first base with a Phred score <20, and those reads shorter than 300 bp were discarded. High-quality reads were analyzed by using QIIME 1.9.1 software (Caporaso et al., 2010). Briefly, OTUs (Operational Taxonomic Units) were picked at 97% similarity level using a de novo approach, and *uclust* method and taxonomic assignment was obtained by using the RDP classifier and the Greengenes database, following a previously reported pipeline (Berni Canani et al., 2017). Raw sequencing reads were uploaded to Sequence Read Archive (SRA^1^) and are stored under project accession number PRJNA649740. To avoid biases due to the different sequencing depth, OTU tables were rarefied to the lowest number of sequences per sample. Alpha-diversity analysis was carried out in QIIME on rarefied OTU tables. Evenness index was calculated as Pielou’s Evenness index J’, as reported in: http://scikit-bio.org/docs/latest/generated/skbio.diversity.alpha.html.

The taxonomy tables were imported into the R software^2^ for statistical analyses and visualization.

### LH-PCR and Calculation of Diversity Indices

In order to estimate which bacterial species were still present in the cheeses at different ripening stages and which underwent lysis during ripening, LH-PCR was performed on both whole and lysed fractions of cheese samples. The primer pair 63F 5′ end labeled with 6-carboxy-fluorescein (6-FAM) and 355R was used as described by Lazzi et al. (2004). The Domain A of the variable region of the 16S rRNA gene was analyzed. Reaction, amplification, and capillary electrophoresis conditions were the same as those used by Bottari et al. (2010). The fragment sizes (base pairs) were determined using GeneMapper software version 4.0 (Applied Biosystems, Foster City, United States), a local Southern method to generate a sizing curve from the fragment migration of the internal size standard (GS500 LIZ^®^; Applied Biosystems, Foster City, United States) and a threshold of 150 fluorescence units. Each peak, corresponding to amplicon of specific length on the electropherogram profile, was attributed to bacterial species according to published databases (Lazzi et al., 2004; Gatti et al., 2008), and the areas under the recognized peaks were used to estimate the amount of the assigned species in the samples. Total area under all the peaks (sum of attributed and unattributed peaks) of the LH-PCR electropherograms was used for measuring the total amount of DNA arising from both intact and lysed cells (D’Incecco et al., 2016). Ecological indices throughout ripening were calculated based on LH-PCR results. Diversity indices (Shannon and Simpson) were calculated as follows: Shannon index, H = −Σpiln(pi), and Simpson index, D = Σpi2 where pi is the ratio between the area of each peak and the sum of all peak areas in the sample. The Simpson’s index value is given as 1 – D, given that this way of presenting it means that a higher value reflects higher diversity. Richness (S = the number of species) and Evenness (E = H/Hmax; Hmax = ln S) were also calculated. For each matrix, the mean value of six samples coming from different dairies are shown and standard errors were calculated.

### Cheese Water Soluble Extract

Ten grams of finely grated cheese were suspended in 45 ml of 0.1 N HCl. (L,L)-phenylalanyl-phenylalanine (Phe-Phe) was added as an internal standard (2.5 ml of a 1 mM solution). The suspension was homogenized for 1 min using an Ultra-Turrax homogenizer (IKA Werke GmbH & Co. KG, Staufen, Germany) and then centrifuged at 3,400 × g for 30 min at 4°C. The solution was filtered through paper filters, and extracted 3 times with 40 ml of ethyl ether. Diethyl ether residues were removed using a Rotavapor (Buchi, New Castle, DE, United States); then, the aqueous solution was filtered through a 0.45 μm filter. 0.5 ml of 0.1% of formic acid solution were added to 1.5 ml of filtered extract, and the solution was passed through Vivaspin 2 Ultrafilter (Sartorius, Gottingem, Germany) with polyether sulfone (PES) membrane (nominal MWCO 10 kDa) previously washed and conditioned following manufacturer instructions. The filtration lasted for 45 min at 4,930 × g, using a centrifuge Hettich Universal 320R (Kirchlengern, Germany) at 23°C. Further washing steps of the ultrafilters were performed (3 times using acidified water at 0.1% formic acid). Final extracts were dried using a Rotavapor (Buchi, New Castle, DE, United States). The filtrate was dried under nitrogen, dissolved in 250 μL of 0.1% HCOOH in H_2_O, and analyzed by UPLC/ESI-MS. Each sample was extracted and analyzed in triplicate.

### UPLC/ESI-MS Analysis

The peptides were semi-quantified by UPLC/ESI-MS through comparison with Phe-Phe as internal standard, as described in Bottari et al. (2017) with the following modifications: no pre column was used, sample temperature: 6°C, injection volume: 2 μL, source temperature: 100°C, desolvation temperature 150°C, scan duration: 1 s.

The peptide fraction analysis yielded a TIC (total ion chromatogram) for each sample. For each signal of interest, the most intense ions were extracted, obtaining a XIC (extract ion chromatogram). The area underlying the peaks was then determined. The peptides were selected according to the signal intensity and the trend (increasing or decreasing) during ripening. Molecular masses of the most abundant peptides were obtained by analyzing the mass spectra associated with the most intense chromatographic peaks. One hundred and eighty-nine different peptides were first considered in the preliminary screening, with molecular masses ranging from about 200 to more than 7,000 Da. Peptides were then semi quantified in all the samples against the internal standard Phe-Phe according to a method previously reported (Sforza et al., 2003, 2004, 2012). Only peptides that gave a minimum chromatographic signal corresponding to 20% of the signal of the internal standard in at least one sample were considered for sequence identification. Thus, starting from the original set of 189 peptides, 34 peptides most representative of the cheese peptide profile were selected for sequence analysis.

### Statistical Analysis

Statistical analyses were carried out using IBM SPSS Statistics software (version 26.0, Armonk, NY, United States). Kaiser-Meyer-Olkin measure of adequacy of sampling: 0.718. Bartlett’s sphericity test sign.: 0.000. Data linearity was assessed both with Kolmogorov-Smirnov and Shapiro-Wilk tests: 38 out of 50 variables were not normally distributed. Then, bivariate correlation was performed using Spearman’s coefficients, with a two-tailed significance test, and pairwise case exclusion for missing values. Significance was fixed to a *p* < 0.05. The ANOVA followed by the Tukey HSD test were performed to detect statistical differences (*p* ≤ 0.05) among microbial counts and biodiversity indices as a function of ripening time.

SIMCA 16.0.1 (Sartorius Stedim Data Analytics, Göttingen, Germany) software was used to create principal component analysis (PCA) biplot to get a visual interpretation of the analyzed data.

## Results

### Bacterial Dynamics During PR Cheese Ripening

Sixty-nine PR cheese samples (only 67 could be successfully sequenced) were analyzed by 16S rRNA gene amplicon high-throughput sequencing. A total of 3,344,483 raw reads were obtained after the sequencing step, of which 3,168,658 passed the filtering steps, with an average number of 28,034 reads/sample. The number of OTUs, the estimated sample coverage (ESC), as well as species richness (Chao1 indices) and diversity (Simpson, Shannon and Evenness indices) indicators were calculated for all samples after the rarefaction step and are reported in Supplementary Table 1. Good’s coverage indicated that for all samples more than 99% of the bacterial diversity was described, and species richness ranged from a minimum of 24 OTUs in a 1-month-old sample to a maximum of 142 OTUs in 24-month-old samples. Diversity indices were considered according to the selected sampling times and are represented in Supplementary Figure 1, given that they are in good agreement with diversity indices calculated on the LH-PCR profiles.

**FIGURE 2:**
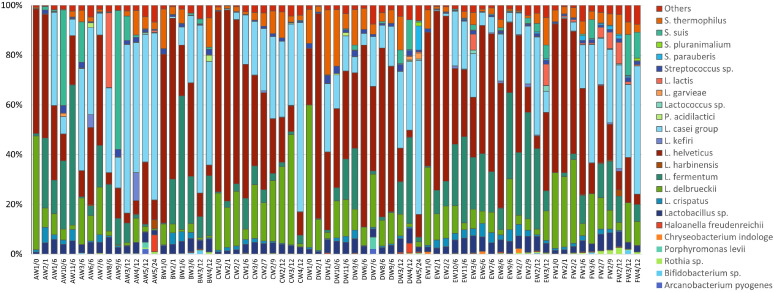
The relative abundance of OTUs based on 16S rRNA sequencing; only bacterial species present in at least two samples with an abundance above 1% are shown. Results are reported for dairies A to F, samples are named according to the scheme reported in section “Cheese Sampling.”

Sequences were assigned to 21 different phyla, among which *Firmicutes* (97.4%), *Actinobacteria* (0.8%), *Proteobacteria* (0.6%), and *Bacteroidetes* (0.6%) represented between 97.9 and 100% of the bacterial population. Among *Firmicutes*, Bacilli are by far the most represented class, with values ranging between 86.5 and 99.9% of the entire microbiota. Only 23 OTUs were present in at least two samples with an abundance above 1% (Supplementary Table 2), and, among these, only 6 OTUs were present in at least 50% of the samples. These are the species *Lactobacillus helveticus*, *Lactobacillus delbrueckii*, *Lacticaseibacillus (formerly L. casei* group, Zheng et al., 2020), *Lactobacillus fermentum*, *Streptococcus thermophilus* and *Lactobacillus crispatus*, representing from a minimum of 82.5% to a maximum of 98.6% of the microbiota of the cheese samples (Figure 2).

The starter species, i.e., *L. helveticus* and *L. delbrueckii*, dominated through the first stages of fermentation, representing among 76.3–95.7% of the microbiota after the molding step. After the brining of the curd, these species represented between 57.4 and 94.8% of the entire bacterial population, and, in 2-month-old cheeses, these values ranged between 79.3 and 88.0%. In the subsequent sampling points, these species showed a general decrease in abundances. In the majority of samples (59 samples out of 67), the relative abundance of *L. helveticus* was greater than that of *L. delbrueckii* (in a dairy-independent way).

For all the dairies, the species belonging to *Lacticaseibacillus* were present at low abundances in the first cheese making steps (≤1% in samples between 0 and 2 month) and showed an increase from 6 months of ripening, with average values of 21.7 ± 13.9% (mean ± SD). Their relative abundance further increased after 12 months of ripening (43.2 ± 18.1%) and reached highest values in 24-month-old samples (64.5 ± 3.6%). *L. fermentum* was present in most samples and showed dynamics similar to those of *Lacticaseibacillus*, with an average relative abundance of 16.1 ± 17.7% in 6-month-old samples and 10.3 ± 11.3% in 12-month-old samples. *S. thermophilus* was also detected in the majority of samples, with values ranging from less than 0.1% up to 26%, such as in the case of *Streptococcus suis*, which ranged from less than 0.1% up to 55.7% but with high values found only in few samples from dairies A and F. Another frequent species was *L. crispatus*, which was present at low abundances (from less than 0.1% up to 6.2%), and which showed small increases in 1 month samples (until after 6–7 months of ripening) and decreases afterward. Interestingly, members of the family *Bifidobacteriaceae* were present with abundances lower than 0.1% in most samples, especially in the early cheese-making steps, reaching abundances of 1.4 and 1.6% in 12 month samples from dairies B and F, respectively.

### Viable Cell Counts and Dynamics of Whole and Lysed Bacterial Cells

**FIGURE 3:**
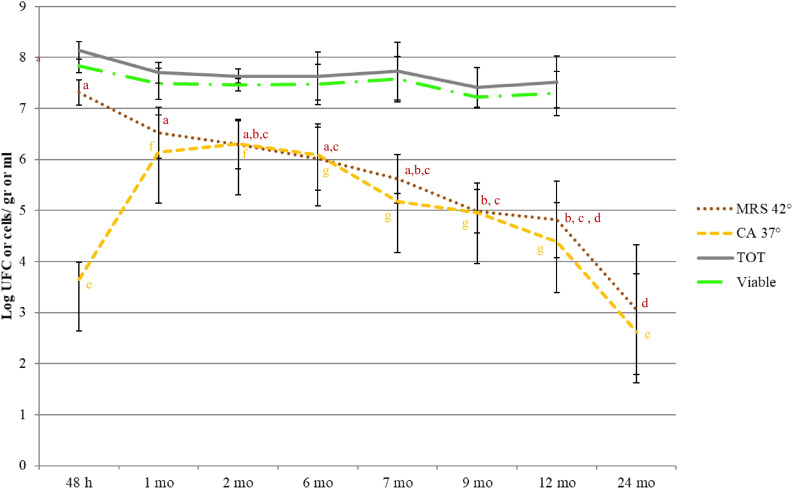
Microbial counts (MRS 42°C: SLAB; CA 37°C: NSLAB; Total cells, Viable Cells) during cheese ripening. For each ripening stage, the means of all the samples with same aging time are shown. Bars represent differences among samples from different dairies (standard errors). Significant differences (*p* < 0.05; ANOVA followed by Tukey *post hoc* test) as a function of the ripening time are indicated by different letters. The coloring of descriptive statistics corresponds with the colors of the variables. The viable and total counts were not significantly different.

**FIGURE 4:**
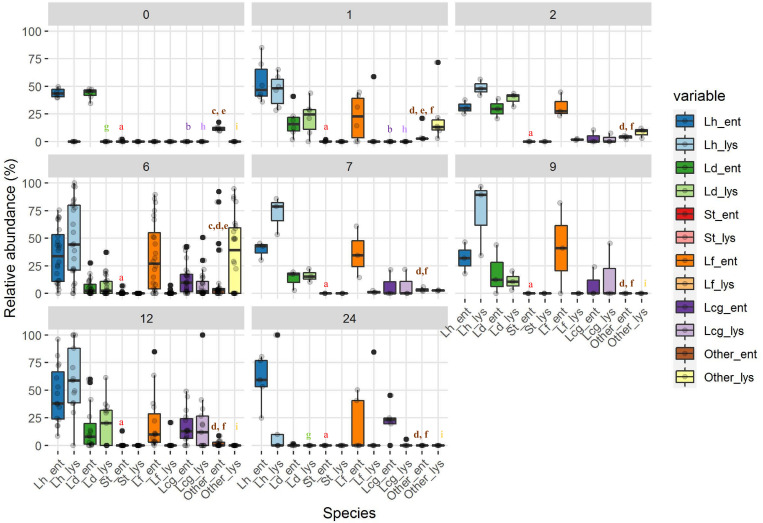
The relative abundance of entire (indicated by _ent) and lysed (indicated by _lys) bacterial cells at different ripening stages. Ripening times are as follows: 0, curd samples, 1, 1 month samples; 2, 2 month samples; 7, 7 month samples; 6, 6 month samples; 9, 9 month samples,12, 12 month samples; 24, 24 month samples. Bacterial species are abbreviated as follows: Lh, *L. helveticus*; Ld, *L. delbrueckii*; St, *S. thermophilus*; Lf, *L. fermentum*; Lcg, *Lacticaseibacillus* (formerly *L. casei* group*).* Significant differences (*p* < 0.05; ANOVA followed by Tukey *post hoc* test) for each species, entire/lysed, as a function of the ripening time are indicated by different letters. The coloring of descriptive statistics corresponds with the colors of the variables. For samples that were statistically different from all the others, no letters were reported.

**FIGURE 5:**
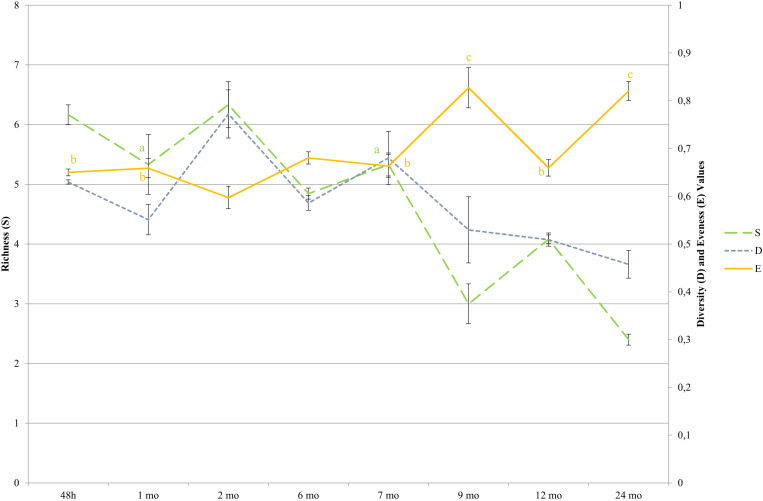
Diversity indices during cheese ripening determined by LH-PCR. Species were detected by comparing the amplicon lengths with the LH-PCR databases. For each ripening stage, the means of all the samples with same aging times are shown. Bars represent differences among samples from different dairies (standard errors). Simpson (D = Σ pi2); the Simpson’s index value is given as 1-D. pi is the relative abundance of a given LH-PCR peak; Richness (S) is equal to the number of species. Evenness (E) is the relative abundance with which each species is represented, [E = H/Hmax; were H is Shannon index (H = −Σ piln(pi)) and Hmax = lnS]. pi is the relative abundance of a given LH-PCR peak and is obtained by dividing the area of each peak with the total area of all peaks in the electropherogram profile for each sample. Significant differences (*p* < 0.05; ANOVA followed by Tukey *post hoc* test) as a function of the ripening time are indicated by different letters for each index. Simpson values were significantly different among all ripening stages, thus no letters were indicated.

Bacterial profiles of 69 PR cheese samples were also described by means of culture-dependent and -independent approaches. Total, viable, and LAB counts are shown in Figure 3. For each ripening stage, the mean values of six samples coming from different dairies are shown. The bars represent the variability among cheeses from different dairies, having been calculated as standard error. Microbial counts that showed a general trend were SLAB (counted on MRS at 42°C) decreased since the first cheese-making steps. The number of NSLAB (counted on CA at 37°C) began, instead, to increase, starting from the curds up to 6 months of ripening stage, and decreasing thereafter. Viable cells counts were in good agreement with culture-dependent microbial counts. Species distribution revealed by LH-PCR was found to be variable, both among different dairies and within a single dairy, at different ripening times (Figure 4). In the early stages, and particularly for 48 h curd samples, a higher relative abundance of SLAB species, such as *L. helveticus* and *L. delbrueckii*, was observed. Longer ripened cheeses, from the sixth month of maturation, were found to contain mainly NSLAB species, such as *Lacticaseibacillus*. As far as the lysed cells fraction was concerned, LH-PCR confirmed that the species undergoing lysis are differently represented both in different dairies and during different amounts of time (Figure 4). From the early stages of cheese ripening, the lysis of both SLAB, such as *L. delbrueckii* and *L. helveticus*, and NSLAB, such as *Lacticaseibacillus* species, was observed. Figure 5 presents the ecological indices during manufacture and ripening, calculated from LH-PCR results, to evaluate the microbial diversity of cheeses at different stages of ripening for the six cheese manufacturing processes. For each ripening stage, the mean value of all the samples with the same ripening times coming from different dairies are shown. The bars represent the variability among cheeses from different dairies, having been calculated as standard error. The graph includes the totality of the data relating to the presence of microbial species in function of both aging time and different dairy origin, regardless of the number of considered wheels. Among cheese ecosystems, diversity (D), Evenness (E) and Richness (S) showed changing trends, with an increase during the first two months and, then, a gradual decrease during ripening. The highest number of species in the community (S) was observed in 2-month-old cheeses, while major differences among dairies were revealed at the beginning of cheese ripening (among 1 and 2-month-old samples) and among 7 and 9-months-old samples. Both microbial diversity and differences among dairies decreased throughout the ripening time.

### Peptide Composition

**FIGURE 6:**
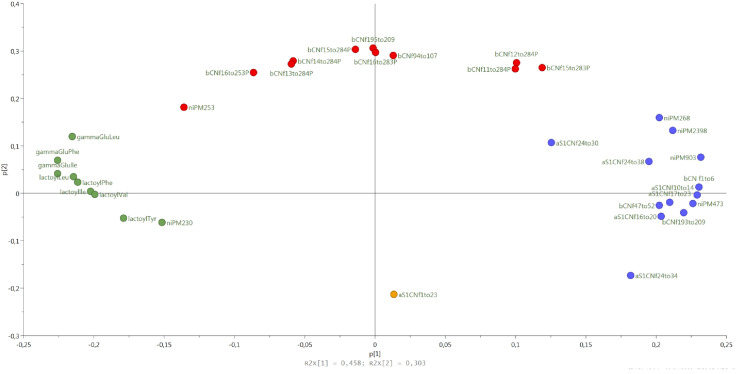
The loading plot of the peptides semi-quantitative data colored according to the aging time: 48 h (yellow), 1–2months (blue), 6 months (red), 7–24 months (green).

Semi-quantitative data for all peptides were used as variables in a principal component analysis (PCA). The loading plot of the PCA is reported in Figure 6. Peptides clustered into 4 groups according to ripening stages, from curd to 24-month-old cheeses. Samples classified according to dairy of production did not cluster separately on the score plot (data not shown), indicating that the production in different dairies was not significantly responsible for peptide variability. A first cluster, corresponding to curd samples, was characterized by one peptide with a molecular weight of 2,763 Da, identified as a fraction of α_s1_ casein (CN), α_s1_-CNf(1–23). A second cluster, corresponding to 1 and 2-month-old cheese samples, was characterized by the following peptides: α_s1_-CNf(24–34), α_s1_-CNf(16–20), α_s1_-CNf(24–38), α_s1_-CNf(24–30), α_s1_-CNf(17–23), α_s1_-CNf(10–14), β-CNf(47–52), β-CNf(193–209), β-CNf(1–6) and few non-identified peptides. A third cluster, corresponding to 6-month-old cheese samples was characterized by the presence of β-CNf(15–28)3P, β-CNf(12–28)4P, β-CNf(11–28)4P, β-CNf(94–107), β-CNf(195–209), β CNf(16–28)4P, β CNf(15–28)4P, β CNf(14–28)4P, β CNf(16–25)3P, β CNf(13–28)4P, and one non-identified peptide. These peptides are phospho-peptides, arising from β – casein, hydrolyzed in position 28–29 or 25–26, followed by further degradation starting from the N-terminal. The endopeptidase action is the result of endopeptidases with specificity for basic residues, given that the amino acid in position 25 and 28 is always the lysine. A final cluster, corresponding to the longer ripened cheeses (7, 9, 12, and 24 months), was characterized by the presence of NPAD (Non-Proteolytic Aminoacyl Derivatives), namely gamma- glutamyl-, lactoyl-, and pyro glutamyl-amino acids.

### The Correlation Between Cheese Microbiota and Peptide Composition

The clustering of cheese samples according to both their microbial composition and the measured peptide fraction showed the existence of a ripening trend among the variables (Supplementary Figure 2). Cheese samples at 0, 1 or 2 months of ripening showed partly overlapping microbial composition and clustered with peptides n.i. PM473, β-CN f(193–209), β-CN f(1–6), β-CN f(47–52), α_s1_-CN (10–14), α_s1_-CN f(16–20), α_s1_-CN f(17–23), and α_s1_-CN f(24–34), which correspond to early peptide products from LAB proteolytic activity. At 6, 7, and 9 months of ripening, the bacterial composition of the samples was more variable, while the peptide fraction showed an evolution from early proteolysis products toward smaller fragments (Supplementary Figure 2). Longer ripened PR cheese samples (12 and 24 months) retained some variability in terms of microbial composition but were characterized by the accumulation of NPADs.

**FIGURE 7:**
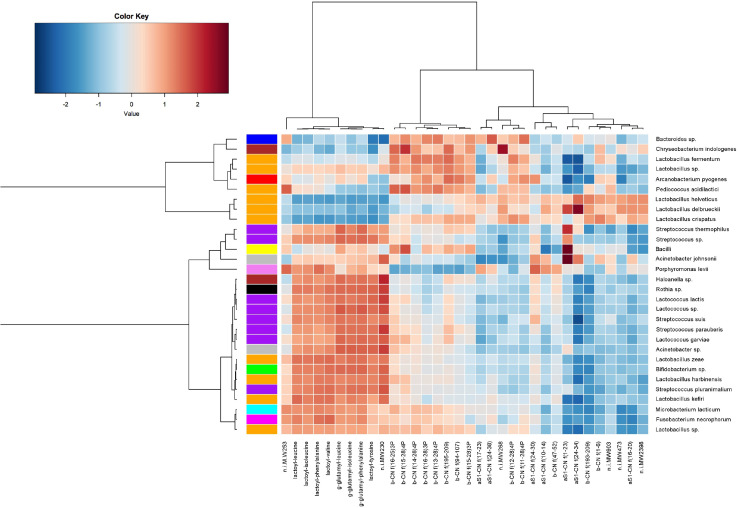
A heat map showing Spearman’s correlation between cheese microbiota and peptides abundance. Rows and columns are clustered by Euclidean distance and Ward linkage hierarchical clustering. The intensity of the colors represents the degree of association, as measured by Spearman correlations. Only taxa occurring in at least 20% of the samples were included.

To visualize correlations existing between specific microbial taxa and proteolytic products, a heat map was built. As shown in Figure 7, proteolytic derivatives of caseins formed three separate clusters: a first cluster (A) is characterized by early ripening stage peptides and shows high correlation with taxa belonging to SLAB group, such as *L. helveticus* and *L. delbrueckii*, along with the less abundant species *L. crispatus*. Cluster B, characterized by proteolytic derivatives of β-caseins, showed a positive correlation with species that develop during the intermediate phase of the ripening, such as *L. fermentum* or *Pediococcus acidilactis*. The last cluster (C) was characterized by NPADs, that correlated with non-starter taxa, such as the *Lacticaseibacillus*, *Lentilactobacillus kefiri*, *Lactobacillus harbinensis*, and *Bifidobacterium* spp. The species *Lactococcus lactis* and *S. thermophilus* were also positively correlated with an increase in abundance of these compounds at later ripening stages.

**FIGURE 8:**
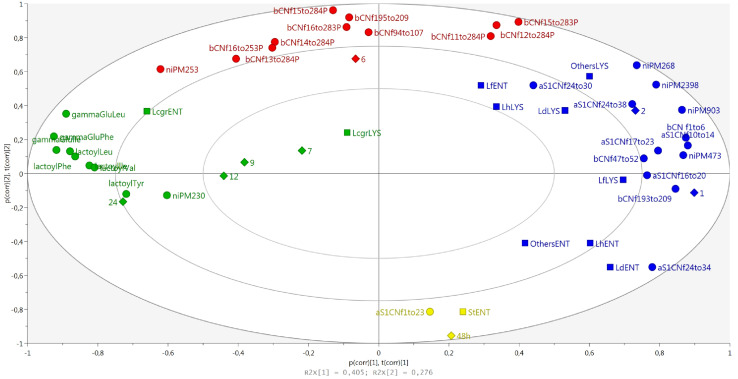
The biplot of the distribution of microbial species and peptides according to the aging times and colored according to marked groups.

LH-PCR data in cheeses at different ripening times were combined with all the identified peptides to evaluate possible correlations between the dynamics of whole and lysed bacterial cells and peptide composition from curd to 24-month-old cheeses (Figure 8). DNA from entire and lysed bacterial cells and peptides were grouped according to ripening time. In particular, the 4 groups, highlighted by different colors, showed a counterclockwise trend in the samples, starting from 48 h to 24 months of ripening. From this analysis, it was possible to observe that the succession of microbial species during ripening was accompanied by a marked change in peptide composition. As expected, a first cluster corresponding to the 48 h curd samples was characterized by the presence of α_S1_-CNf(1–23) that derives from the first proteolysis stages occurring at the beginning of ripening, and the presence of entire cells of *S. thermophilus*, a minority species found in curd samples (Figure 4). The second cluster, corresponding to 1 and 2 month ripened cheeses, grouped together the peptides deriving from the first proteolysis stages and the entire and lysed microbial cells of *L. helveticus*, *L. delbrueckii*, and *L. fermentum*. The third cluster (6-month-old\cheeses) was characterized mostly by the presence of phospho-peptides arising from β- casein. Finally, the fourth cluster (7 to 24-month-old cheeses) was characterized by NPAD and *Lacticaseibacillus*, both entire and lysed cells.

## Discussion

The LAB viable count in the 69 PR samples analyzed in the present study showed a reduction of SLAB viability within the first month of ripening along with the NSLAB count increase. This is in good agreement with Gatti et al. (2014) who reported that in long ripened cheeses, such as PR, SLAB decrease within a few hours or a few days from the start of the cheese-making process, while NSLAB grow slowly and become the dominant microbiota in the ripened cheese. SLAB, in fact, are known to be abundantly present in the first stages of PR cheese making. They originate mainly from the natural whey starter and actively metabolize the lactose of milk (Gatti et al., 2014). Later, during ripening, when the environment becomes unfavorable due to low sugars, whey drainage, and increasing salt, NSLAB dominate thanks to their ability to use energy sources other than lactose and resistance to environmental stresses (Sgarbi et al., 2014). To get a deeper insight into the bacterial community composition and dynamics of the analyzed cheese samples, HTS and LH-PCR were used. Results obtained by amplicon sequencing showed that, despite the fact that the cheeses from different dairies exhibit a certain degree of variability, the composition of the bacterial population is characterized by the interplay of different LAB and is shaped by technological processes, as observed by other authors (Gatti et al., 2014; De Filippis et al., 2016; Gobbetti et al., 2018). Curds from all dairies were, in fact, dominated by the species *L. helveticus* and *L. delbrueckii*, which derive from the natural whey starter used for cheese making (De Filippis et al., 2014; Bertani et al., 2020). After the brining step (1-month-old samples and later), a compositional change occurs in cheese microbiota that shows a decrease in the relative abundance of the SLAB species, while NSLAB population, initially present at low abundances, find suitable conditions for development (Lazzi et al., 2014; Bottari et al., 2018). Indeed, after 6 months of ripening, cheeses from all dairies showed an increase of *Lacticaseibacillus*, which is known to dominate the microbiota of PR cheese during ripening (Gatti et al., 2014). In the 6-month-old samples, other minority species, such as *L. fermentum*, *L. crispatus*, *S. thermophilus* and *Lc. lactis*, were also present, as observed in other long-ripened, raw milk cheeses manufactured with similar technology (Alessandria et al., 2016; Levante et al., 2017).

While amplicon sequencing was performed on total DNA extracted from cheeses, LH-PCR allowed semi-quantification of DNA extracted from entire cells, to estimate which bacterial species were still present in the cheeses at different ripening stages, and from lysed cells, permitting the estimation of which LAB underwent lysis during ripening (Gatti et al., 2008). This allowed to assess the progress of cell lysis as a function of time and the most affected microbial species. This aspect is of major importance for the evaluation of cheese ripening. In fact, after cell lysis, bacteria release intracellular enzymes important for proteolysis, thus for cheese maturation. The greater the number of growing cells, the greater the subsequent cell lysis and the number of released intracellular enzymes (D’Incecco et al., 2016; Lazzi et al., 2016). The presence of whole cells of *L. helveticus* and *L. delbrueckii* in curd samples confirmed what was previously observed, as these species come mainly from the natural whey starter and are frequently found at the beginning of ripening (Bottari et al., 2013; Gatti et al., 2014). The lysis of both SLAB and NSLAB observed since the early stages of cheese-making, confirmed that cells undergo autolysis during cheese ripening due to stressful environment conditions. The observation of an autolysis affecting SLAB first, and NSLAB at a later ripening stage, sustains the hypothesis that NSLAB can better survive to those conditions (Gatti et al., 2014). Biodiversity indices indicated that cheese curds were characterized by a high level of richness, followed by a slight decrease after brining (1 month old samples) and a subsequent increase in 2 month old samples. This is consistent with the growth trend observed by culturing, and could be correlated to the growth of NSLAB reported during the first months of ripening (Gatti et al., 2014). NSLAB in fact, arising mainly from milk, enrich microbial diversity of curd, which is mostly represented by whey SLAB.

Differences, in terms of biodiversity, among the various dairies, were small at curd stage. This is likely due to the prevalence of SLAB coming from the starter, which, although prepared differently by each dairy, generally contains mainly *L. helveticus* and *L. delbrueckii* and possibly *S. thermophilus* and *L. fermentum* (Bottari et al., 2010, 2013; De Filippis et al., 2014; Bertani et al., 2020). Major differences revealed among dairies between 1 and 2 months old samples and 7 and 9 months old samples, are consistent with the growth of NSLAB coming mainly from raw milk and their higher adaptability to specific environmental conditions, driven by cheese making parameters (Bottari et al., 2018). In the following ripening stages, biodiversity indices presented some differences according to the investigation method: while metataxonomic data showed a consistent increase of all the indicators until 9 months of ripening, LH-PCR performed on entire bacterial cells indicates that there is a decrease in richness and diversity after 2 months of ripening. This is consistent with the decrease of viable counts of both SLAB and NSLAB in longer ripened cheeses, and with the bacterial lysis occurring at higher extent after this time point. This ripening stage was previously reported as crucial for the microbial dynamics and diversity of long ripened raw milk cheeses (Santarelli et al., 2013). In the later ripening stages, both differences among dairies and the biodiversity indices showed a decrease. This is justified by microbial selection, inevitably occurring along maturation, which allows the only growth of those microbes that can use energy sources other than milk carbohydrates and tolerate decreasing a_*w*_ value (Gatti et al., 2014). This finding is in agreement with the isolation of few species from long ripened PR (Gatti et al., 2008) and is further confirmed by the E index, calculated from the LH-PCR data on entire cells in the present study, which increased over time and precisely indicates the predominance of one or a few species compared to the total.

Peptide analysis is of utmost importance in determining cheese quality and ripening. The peptide fraction of cheese is, indeed, a direct consequence of the proteolytic events occurring during cheese ripening. Along with cheese aging, milk and bacterial proteases cleave caseins into shorter peptides, which thus accumulate in the cheese, reaching a maximum, and then decreasing due to their own degradation into shorter fragments. An exception to this phenomenon is NPADs, which are not degraded and continue to accumulate. Given the production process of a cheese, the formation of specific peptides (or peptide classes) is typical of certain stages of maturation, so they can be used as molecular markers of aging time (Sforza et al., 2012). Proteolysis also strongly influences the quality of the cheese, affecting the texture (high proteolysis correspond to soft texture), the taste (peptides are usually bitter, with the exception of NPADs which are kokumi), the flavor (amino acids are precursors of aromas), the digestibility (high proteolysis improves digestibility), allergenicity (some allergens can be degraded into non-allergenic peptides), and so on (Sousa et al., 2001; Toelstede et al., 2009; Alessandri et al., 2012). It is thus clear that analyzing the peptide fraction can provide a lot of guidance in assessing cheese quality and maturation. The differences in the microbiota (especially for the NSLAB group) of different dairies could lead to a different peptide profile due to a different kinetic and action of the proteolytic enzymes. However, analyzing samples from 48 h curd up to 24 months of aging, we found that the huge peptide evolution occurring during ripening greatly overpowered the possible small differences among cheeses from different dairies with the same aging time. The clustering of cheese samples confirmed that ripening stage is the characteristic that most affects the observed cheese peptide variability, fully confirming the data reported in Sforza et al. (2012). More specifically, four peptide groups were clustered according to the ripening stage: cluster 1 (curd, 48 h), cluster 2 (from 1 to 2 months of aging), cluster 3 (6 monthsof aging), and cluster 4 (from 7 months to 24 months of aging). Peptides typical of the earliest stages of ripening (48 h curds and 1-month-old cheeses) derive from the first proteolysis stages occurring at the beginning of ripening, when the action of chymosin on α_s1_-CN generates the fragments available for the subsequent LAB proteolytic action. The first peptide to be formed derives from the cleavage of the peptide bond between the 23th and the 24th amino acid residue of α_s1_ casein, and constitute the N-terminal of the protein (α_s1_-CN f1–23). This peptide is produced in the very early stages of cheese making by the action of chymosin (more thermolabile) and cathepsin D (more thermostable) (Gagnaire et al., 2011). Then, in the two months that follow, the peptide α_s1–_CN f1–23 is broken into smaller fragments (α_s1_ CN f10–14, α_s1_-CN f16–20, and α_s1_-CN f17–23). As previously observed in other cheeses (e.g., Emmental) (Gagnaire et al., 2001), the N-terminal sequence of α_s1–_casein is proteolyzed into several peptides (in addition to the aforementioned ones, also α_s1_-CN f24–30, α_s1_-CN f24–34, and α_s1_-CN f24–38). On the contrary, no peptides coming from the C-terminal of the protein were detected, meaning that this part of the protein remains intact and is not attacked by proteases, neither endogenous of the milk nor microbial. In this second cluster (corresponding to the first two months of aging), β-casein peptides also appear. Differently from what observed for α_s1_ casein, in β-casein, peptides deriving both from the N-terminal and the C-terminal were identified (β CN f1–6 and β CN f 193–209). The formation of the peptide β-CN f(1–6) can be ascribed to the action of cell-envelope proteinases from thermophilic *Lactobacillus* (Gagnaire et al., 2001). The β-CN f 193–209 is generated by the cleavage of the peptide bond between Leu192-Tyr193, which can be hydrolyzed both by cathepsin D and cell-envelope proteinase of starter bacteria (*Lactobacillus* genus, mainly) (Gagnaire et al., 2001). The third cluster mainly contains phosphopeptides, and all of them derive from the region 11-28 of β-casein, in agreement with previous work (Sforza et al., 2003). These phosphopeptides share a common feature: the cleavage site in 28th position; plasmin can cleave the peptide bond at Lys28-Lys29 of β-casein (Lund and Ardö, 2004), but thermophilic starter bacteria may play an important role, as well. Finally, the fourth cluster contains NPADs, which accumulate during ripening since their chemical structure is not recognized by the proteolytic enzymes. Interestingly, the abundance of α_S1_-CN f(1–23) – characteristic of the first stages of cheese manufacturing, within 48 h, thus derived from the proteolytic actions happening in the curd – is positively correlated with the total amount of DNA from entire cells of *L. helveticus* and *L. delbrueckii*, two species typical of PR natural whey starters (Bottari et al., 2010, 2013; De Filippis et al., 2014; Bertani et al., 2020). Indeed, SLAB develop mainly during the first days of PR aging, when lactose is still available for their metabolism (Gatti et al., 2014). Peptides found in the 6-month-old cheese samples are phospho-peptides deriving from β-CN. Their significant correlation with microbial counts on CA at 37°C is particularly interesting because CA is the medium in which NSLAB grow, meaning that degradation of β-CN to short polypeptides can mainly be attributed to NSLAB microbiota, as it has been previously hypothesized (Sforza et al., 2012). NSLAB mainly develop after SLAB, and, consistently, the amount of most β-CN peptides rises after α_s1_-CN peptides. Peptide β-CN 193–209 (the C-terminal part of β casein), one of the most representative of this class, starts to accumulate at the beginning of cheese aging. Likewise, NPADs accumulate during aging, but they start to increase later and for up to 24 months. Thus, the trend of most of the β-CN peptides is not related with that of NPADs, while these latter are very strongly related to each other because they accumulate together during ripening, reaching their maximum amount at the end of cheese aging (Sforza et al., 2009). The accumulation of such molecules is probably due to their uniquely unconventional structure, which prevents the hydrolysis by exopeptidases. The enzymatic systems involved in their production has been reported to be likely intracellular and released after LAB death (Sforza et al., 2012; Bottesini et al., 2014). In fact, NPADs revealed a negative significant correlation with all the microbial counts with the exception of the amount of DNA from entire cells of *Lacticaseibacillus*, the dominant species in PR cheese up to 20 months of ripening (Neviani et al., 2009). This species could be responsible for the accumulation of these peptides, potentially involved in flavor formation. In addition to *Lacticaseibacillus*, other minor species from the NSLAB moiety might contribute to this accumulation, such as *Len. kefiri*, *L. harbinensis*, and *Bifidobacterium* spp. These results would sustain other findings regarding this very interesting topic (Sgarbi et al., 2013, 2014).

The aim of the research was to explore the link between the PR microbiota and the proteolysis that occurs during ripening, which is the basis for the valuable and recognizable characteristics of long-ripened cheeses. The complex organization of the sampling plan and the application of different analytical and statistical analyses enabled the conclusions and hypothesis made in previous works regarding the relation among PR microbiota’s composition and dynamics and peptide evolution to be strengthened and confirmed. The highlight of this research was due to the combined approach of HTS and LH-PCR on both entire and lysed cells to evaluate their impact on proteolysis. The consequence of the bacterial presence and activity of cells and their released enzymes is not limited to the protein component of this widely appreciated cheese but also to the lipid component of the partially skimmed milk and the degradation of sugars, as well as minor components. However, the interplay of LAB and proteins has to be considered as the most relevant feature of this type of cheese. By relating the peptide evolution of cheeses of different dairies to specific microbial composition at defined times, we were able to expand on what was previously known about the most important aspects of cheese ripening.

Samples from different dairies were characterized by quite similar microbiota at curd level, due to both raw milk and natural whey starters’ microbiota, but later, during ripening, the microbial composition evolved, revealing major differences among dairies between 1 and 2-month-old samples and 7 and 9-month-old samples. This differences were mainly due to the NSLAB species, which are more related to different peptide profiles given the different kinetics and activities of the proteolytic enzymes. With this observation, we highlighted once again the major role of NSLAB in ripened cheese proteolysis.

As it is widely known, PR is produced under strict regulations, although it is, nevertheless, a high quality, artisanal cheese, with uniquely appreciated features that can vary. The awareness of the association between the microbial composition (and evolution) and the proteolysis level (linked to the peptide composition), would be of practical interest for PR producers (and, more generally, for cheese producers), as the knowledge of this phenomenon allows one to monitor the cheese ripening and take corrective action in time to obtain the desired quality attributes. Moreover, the potential use of several peptides as markers of a specific microbial composition affords the possibility of taking advantage of it to protect and valorize the specificity and connection of PR cheese to its production territory.

## Data Availability Statement

The datasets presented in this study can be found in online repositories. The names of the repository/repositories and accession number(s) can be found below: https://www.ncbi.nlm.nih.gov/, sra/PRJNA649740.

## Author Contributions

BB, MN, SS, and MG conceived the work. BB, CB, AL, and FD performed the analyses. BB, MG, SS, FD, and DE interpreted the results. EB, AL, BP, and FD performed statistical analyses. BB, EB, AL, BP, and CB drafted the original manuscript. EB, AL, and FD prepared figures and tables. BB, SS, MN, MG, BP, FD, and DE critically revised the manuscript. All authors contributed to the article and approved the submitted version.

## Conflict of Interest

MN was employed by the company Consorzio del Formaggio Parmigiano Reggiano. The remaining authors declare that the research was conducted in the absence of any commercial or financial relationships that could be construed as a potential conflict of interest.

## Footnotes

1 https://www.ncbi.nlm.nih.gov/sra

2 http://www.r-project.org/
