# Supplementary material for: The Interrelationship Between Microbiota and Peptides During Ripening as a Driver for Parmigiano Reggiano Cheese Quality
Source: Front Microbiol. 2020 Oct 2;11:581658. doi: 10.3389/fmicb.2020.581658 (PMC7561718; doi:10.3389/fmicb.2020.581658)
Supplement: Supplementary file 1 [file Table_1.DOCX]

**Supplementary Table 1**: Diversity indices for 16S rRNA amplicons from cheese samples. Data are obtained after multiple (10) rarefaction of samples at 11,000 reads. All data are reported as mean ± S.D.

| Code (FDF) | Code  (BB) | Chao1 | Good's coverage (%) | Observed species | Shannon | Simpson | Evenness |
| --- | --- | --- | --- | --- | --- | --- | --- |
| 1567.F1.0 | AW1/0 | 77.49 ± 17.27 | 99.81 ± 0.03 | 44 ± 8 | 1.36 ± 0.01 | 0.54 ± 0 | 0.36 ± 0 |
| 1567.F2.1 | AW2/1 | 100.96 ± 68.71 | 99.82 ± 0.02 | 35 ± 27 | 2.01 ± 0.01 | 0.66 ± 0 | 0.57 ± 0.01 |
| 1567.F1.6 | AW1/6 | 100.22 ± 18.82 | 99.75 ± 0.04 | 58 ± 28 | 2.36 ± 0.01 | 0.74 ± 0 | 0.58 ± 0.01 |
| 1567.F10.7 | AW10/6 | 99.47 ± 29.31 | 99.76 ± 0.03 | 56 ± 28 | 2.57 ± 0.01 | 0.76 ± 0 | 0.64 ± 0 |
| 1567.F11.6 | AW11/6 | 96.62 ± 18.7 | 99.78 ± 0.03 | 56 ± 27 | 2.13 ± 0.01 | 0.63 ± 0 | 0.53 ± 0.01 |
| 1567.F3.6 | AW3/6 | 159.19 ± 20 | 99.58 ± 0.04 | 93 ± 25 | 2.58 ± 0.02 | 0.76 ± 0 | 0.57 ± 0 |
| 1567.F6.6 | AW6/6 | 139.31 ± 16.87 | 99.62 ± 0.04 | 88 ± 16 | 2.74 ± 0.01 | 0.76 ± 0 | 0.61 ± 0 |
| 1567.F7.6 | AW7/6 | 140.4 ± 34.89 | 99.69 ± 0.04 | 64 ± 18 | 2.38 ± 0.01 | 0.73 ± 0 | 0.57 ± 0.01 |
| 1567.F8.7 | AW8/6 | 103.08 ± 15.88 | 99.77 ± 0.03 | 63 ± 27 | 2.45 ± 0.01 | 0.75 ± 0 | 0.59 ± 0.01 |
| 1567.F9.7 | AW9/6 | 107.18 ± 18.93 | 99.7 ± 0.03 | 67 ± 28 | 2.15 ± 0.01 | 0.65 ± 0 | 0.51 ± 0.01 |
| 1567.F3.12 | AW3/12 | 133.53 ± 16.75 | 99.65 ± 0.03 | 81 ± 27 | 1.91 ± 0.01 | 0.52 ± 0 | 0.43 ± 0 |
| 1567.F4.12 | AW4/12 | 169.3 ± 35.69 | 99.58 ± 0.05 | 95 ± 28 | 2.5 ± 0.02 | 0.69 ± 0 | 0.55 ± 0.01 |
| 1567.F5.12 | AW5/12 | 138.73 ± 24.12 | 99.7 ± 0.02 | 84 ± 15 | 2.41 ± 0.01 | 0.67 ± 0 | 0.55 ± 0 |
| 1567.F5.24 | AW5/24 | 179.93 ± 20.56 | 99.55 ± 0.05 | 123 ± 26 | 2.21 ± 0.01 | 0.54 ± 0 | 0.46 ± 0 |
| 593.F1.0 | BW1/0 | 92.28 ± 29.19 | 99.78 ± 0.05 | 49 ± 27 | 1.6 ± 0.01 | 0.5 ± 0 | 0.41 ± 0.01 |
| 593.F2.1 | BW2/1 | 96.33 ± 72.54 | 99.82 ± 0.04 | 39 ± 27 | 1.75 ± 0.02 | 0.54 ± 0.01 | 0.48 ± 0.01 |
| 593.F1.6 | BW1/6 | 125.79 ± 17.88 | 99.66 ± 0.04 | 76 ± 14 | 2.21 ± 0.02 | 0.66 ± 0 | 0.51 ± 0.01 |
| 593.F3.6 | BW3/6 | 175.2 ± 21.47 | 99.55 ± 0.04 | 108 ± 28 | 2.51 ± 0.02 | 0.74 ± 0 | 0.54 ± 0 |
| 593.F3.12 | BW3/12 | 206.42 ± 31.9 | 99.51 ± 0.07 | 134 ± 24 | 2.1 ± 0.02 | 0.53 ± 0 | 0.43 ± 0 |
| 593.F4.12 | BW4/12 | 184.7 ± 20.37 | 99.55 ± 0.04 | 115 ± 28 | 2.77 ± 0.01 | 0.75 ± 0 | 0.58 ± 0 |
| 105.F1.0 | CW1/0 | 122.52 ± 55.25 | 99.75 ± 0.04 | 51 ± 28 | 1.17 ± 0.01 | 0.42 ± 0 | 0.3 ± 0.01 |
| 105.F2.1 | CW2/1 | 54.9 ± 25.33 | 99.89 ± 0.03 | 24 ± 28 | 1.16 ± 0.01 | 0.38 ± 0 | 0.37 ± 0.02 |
| 105.F2.2 | CW2/2 | 67.92 ± 17.87 | 99.85 ± 0.02 | 34 ± 27 | 1.58 ± 0.01 | 0.52 ± 0 | 0.45 ± 0.01 |
| 105.F1.6 | CW1/6 | 130.85 ± 24.3 | 99.67 ± 0.04 | 79 ± 28 | 2.18 ± 0.02 | 0.65 ± 0 | 0.5 ± 0.01 |
| 105.F3.6 | CW3/6 | 220.38 ± 49.72 | 99.49 ± 0.05 | 104 ± 27 | 2.59 ± 0.01 | 0.77 ± 0 | 0.56 ± 0.01 |
| 105.F2.8 | CW2/7 | 118.26 ± 31.64 | 99.72 ± 0.05 | 68 ± 17 | 2.57 ± 0.01 | 0.76 ± 0 | 0.61 ± 0.01 |
| 105.F2.10 | CW2/9 | 126.95 ± 24.69 | 99.69 ± 0.04 | 66 ± 27 | 2.52 ± 0.01 | 0.77 ± 0 | 0.6 ± 0.01 |
| 105.F2.13 | CW2/12 | 151.4 ± 23.53 | 99.63 ± 0.04 | 74 ± 27 | 2.52 ± 0.01 | 0.77 ± 0 | 0.58 ± 0.01 |
| 105.F3.12 | CW3/12 | 184.47 ± 36.59 | 99.55 ± 0.06 | 109 ± 28 | 2.29 ± 0.01 | 0.68 ± 0 | 0.49 ± 0 |
| 105.F4.12 | CW4/12 | 193.7 ± 39.63 | 99.53 ± 0.08 | 111 ± 28 | 1.62 ± 0.02 | 0.41 ± 0 | 0.34 ± 0 |
| 2209.F1.0 | DW1/0 | 68.48 ± 18.84 | 99.86 ± 0.02 | 38 ± 28 | 1.78 ± 0.01 | 0.6 ± 0 | 0.49 ± 0.01 |
| 2209.F2.1 | DW2/1 | 71.48 ± 33.91 | 99.83 ± 0.04 | 36 ± 27 | 0.96 ± 0.01 | 0.3 ± 0 | 0.27 ± 0.01 |
| 2209.F1.6 | DW1/6 | 119.63 ± 36.37 | 99.72 ± 0.04 | 62 ± 18 | 2.4 ± 0.02 | 0.75 ± 0 | 0.58 ± 0.01 |
| 2209.F10.7 | DW10/6 | 131.68 ± 85.81 | 99.75 ± 0.03 | 54 ± 27 | 2.69 ± 0.02 | 0.8 ± 0 | 0.68 ± 0.01 |
| 2209.F11.7 | DW11/6 | 174.7 ± 26.84 | 99.55 ± 0.05 | 107 ± 26 | 2.89 ± 0.01 | 0.8 ± 0 | 0.62 ± 0.01 |
| 2209.F3.6 | DW3/6 | 142.31 ± 31.62 | 99.64 ± 0.05 | 81 ± 27 | 2.86 ± 0.01 | 0.81 ± 0 | 0.65 ± 0.01 |
| 2209.F6.5 | DW6/6 | 106.01 ± 24.42 | 99.75 ± 0.02 | 48 ± 28 | 1.53 ± 0.02 | 0.45 ± 0.01 | 0.4 ± 0.01 |
| 2209.F7.7 | DW7/6 | 115.58 ± 9.72 | 99.71 ± 0.03 | 69 ± 28 | 2.86 ± 0.01 | 0.79 ± 0 | 0.68 ± 0.01 |
| 2209.F8.7 | DW8/6 | 160.63 ± 40.78 | 99.61 ± 0.05 | 81 ± 28 | 1.86 ± 0.01 | 0.52 ± 0 | 0.42 ± 0.01 |
| 2209.F9.7 | DW9/6 | 142.98 ± 22.24 | 99.63 ± 0.04 | 80 ± 27 | 2.01 ± 0.01 | 0.61 ± 0 | 0.46 ± 0 |
| 2209.F3.12 | DW3/12 | 217.53 ± 53.03 | 99.52 ± 0.05 | 122 ± 27 | 3.22 ± 0.03 | 0.84 ± 0 | 0.67 ± 0.01 |
| 2209.F4.12 | DW4/12 | 184.26 ± 43.63 | 99.56 ± 0.07 | 121 ± 28 | 3.01 ± 0.02 | 0.78 ± 0 | 0.63 ± 0 |
| 2209.F5.24 | DW5/24 | 206.35 ± 23.81 | 99.53 ± 0.06 | 142 ± 28 | 2.49 ± 0.01 | 0.6 ± 0 | 0.5 ± 0 |
| 599.F1.0 | EW1/0 | 105.17 ± 21 | 99.74 ± 0.03 | 59 ± 26 | 1.82 ± 0.01 | 0.61 ± 0 | 0.45 ± 0.01 |
| 599.F2.1 | EW2/1 | 81.03 ± 23.8 | 99.82 ± 0.02 | 42 ± 27 | 1.5 ± 0.01 | 0.46 ± 0 | 0.4 ± 0.01 |
| 599.F2.2 | EW2/2 | 77.65 ± 14.67 | 99.81 ± 0.03 | 44 ± 15 | 1.74 ± 0.02 | 0.53 ± 0 | 0.46 ± 0.01 |
| 599.F10.7 | EW10/6 | 99.44 ± 6.61 | 99.73 ± 0.02 | 67 ± 28 | 2.56 ± 0 | 0.78 ± 0 | 0.61 ± 0 |
| 599.F11.7 | EW11/6 | 88.17 ± 7.22 | 99.77 ± 0.03 | 67 ± 14 | 2.56 ± 0.01 | 0.76 ± 0 | 0.61 ± 0.01 |
| 599.F3.6 | EW3/6 | 129.81 ± 17.66 | 99.67 ± 0.04 | 82 ± 26 | 3.23 ± 0.01 | 0.85 ± 0 | 0.73 ± 0.01 |
| 599.F6.7 | EW6/6 | 98.89 ± 7.01 | 99.72 ± 0.02 | 74 ± 13 | 2.24 ± 0.01 | 0.67 ± 0 | 0.52 ± 0 |
| 599.F7.7 | EW7/6 | 90.21 ± 10.94 | 99.8 ± 0.02 | 47 ± 27 | 2.21 ± 0.01 | 0.65 ± 0 | 0.57 ± 0.01 |
| 599.F8.7 | EW8/6 | 188.12 ± 25.33 | 99.57 ± 0.04 | 100 ± 28 | 2.61 ± 0.02 | 0.7 ± 0 | 0.57 ± 0.01 |
| 599.F9.7 | EW9/6 | 153.91 ± 47.3 | 99.7 ± 0.04 | 65 ± 25 | 2.43 ± 0.01 | 0.75 ± 0 | 0.58 ± 0.01 |
| 599.F2.7 | EW2/7 | 165.27 ± 17.48 | 99.58 ± 0.04 | 91 ± 27 | 2.4 ± 0.01 | 0.7 ± 0 | 0.53 ± 0.01 |
| 599.F2.10 | EW2/9 | 168.5 ± 20.67 | 99.6 ± 0.04 | 92 ± 26 | 2.69 ± 0.01 | 0.74 ± 0 | 0.6 ± 0.01 |
| 599.F2.12 | EW2/12 | 165.87 ± 35.19 | 99.61 ± 0.07 | 104 ± 19 | 2.65 ± 0.01 | 0.73 ± 0 | 0.57 ± 0.01 |
| 599.F3.12 | EW3/12 | 187.91 ± 38.2 | 99.57 ± 0.05 | 111 ± 29 | 3.42 ± 0.02 | 0.85 ± 0 | 0.73 ± 0.01 |
| 700.F1.0 | FW1/0 | 96.68 ± 14.53 | 99.75 ± 0.03 | 61 ± 29 | 1.59 ± 0.01 | 0.55 ± 0 | 0.39 ± 0 |
| 700.F2.11 | FW2/1 | 72.45 ± 7.69 | 99.82 ± 0.02 | 42 ± 26 | 1.57 ± 0.01 | 0.53 ± 0 | 0.42 ± 0.01 |
| 700.F2.2 | FW2/2 | 89.13 ± 17.67 | 99.78 ± 0.03 | 58 ± 3 | 2.03 ± 0.02 | 0.64 ± 0 | 0.5 ± 0.01 |
| 700.F1.6 | FW1/6 | 201.3 ± 24.96 | 99.51 ± 0.05 | 121 ± 29 | 3.01 ± 0.02 | 0.77 ± 0 | 0.63 ± 0.01 |
| 700.F3.6 | FW3/6 | 168.04 ± 28.77 | 99.58 ± 0.06 | 99 ± 28 | 2.49 ± 0.01 | 0.71 ± 0 | 0.54 ± 0.01 |
| 700.F2.7 | FW2/7 | 123.39 ± 16.79 | 99.71 ± 0.04 | 80 ± 26 | 3.09 ± 0.01 | 0.83 ± 0 | 0.71 ± 0.01 |
| 700.F2.9 | FW2/9 | 164.22 ± 37.83 | 99.62 ± 0.06 | 90 ± 28 | 3.21 ± 0.01 | 0.84 ± 0 | 0.71 ± 0.01 |
| 700.F2.12 | FW2/12 | 118.23 ± 15.61 | 99.74 ± 0.04 | 88 ± 28 | 3.16 ± 0.02 | 0.79 ± 0 | 0.71 ± 0.01 |
| 700.F3.12 | FW3/12 | 238.21 ± 52.92 | 99.49 ± 0.05 | 127 ± 29 | 3.15 ± 0.01 | 0.83 ± 0 | 0.65 ± 0.01 |
| 700.F4.15 | FW4/12 | 188.77 ± 27.92 | 99.57 ± 0.04 | 110 ± 27 | 2.78 ± 0.01 | 0.71 ± 0 | 0.59 ± 0 |
