# Supplementary material for: The Interrelationship Between Microbiota and Peptides During Ripening as a Driver for Parmigiano Reggiano Cheese Quality
Source: Front Microbiol. 2020 Oct 2;11:581658. doi: 10.3389/fmicb.2020.581658 (PMC7561718; doi:10.3389/fmicb.2020.581658)
Supplement: Supplementary file 2 [file Table_2.DOCX]

Supplementary Material

**Supplementary Table 2.** Incidence of OTUs detected by pyrosequencing analysis of 16S rRNA of all DNA in samples from six Parmigiano Reggiano dairies (A to F). Only OTUs with a relative abundance higher than 1% in at least two samples are included.

|  | ***Arcanobacterium pyogenes*** | ***Bifidobacterium* sp.** | ***Rothia* sp.** | ***Porphyromonas levii*** | ***Chryseobacterium indologenes*** | ***Haloanella freudenreichii*** | ***Lactobacillus* sp.** | ***L. crispatus*** | ***L. delbrueckii*** | ***L. fermentum*** | ***L. harbinensis*** | ***L. helveticus*** | ***L. kefiri*** | ***L.* *casei* group** | ***Pediococcusacidilactici*** | ***Lactococcus sp.*** | ***L. garvieae*** | ***L. lactis*** | ***Streptococcus* sp.** | ***S. parauberis*** | ***S. pluranimalium*** | ***S. suis*** | ***S. thermophilus*** |
| --- | --- | --- | --- | --- | --- | --- | --- | --- | --- | --- | --- | --- | --- | --- | --- | --- | --- | --- | --- | --- | --- | --- | --- |
| **AW1/0** | 0 | 0.01 | 0.01 | 0 | 0 | 0.01 | 0.79 | 1.01 | 45.69 | 0.95 | 0 | 50.04 | 0 | 0.03 | 0 | 0.01 | 0 | 0.07 | 0.07 | 0.01 | 0 | 0.07 | 0.42 |
| **AW2/1** | 0 | 0 | 0 | 0 | 0 | 0 | 4.64 | 6.2 | 7.72 | 28.12 | 0 | 49.72 | 0 | 0.26 | 0 | 0 | 0 | 0.01 | 1.43 | 0 | 0 | 1.44 | 0.08 |
| **AW1/6** | 0 | 0.03 | 0.02 | 0 | 0 | 0.08 | 5.82 | 1.87 | 8.43 | 13.48 | 0 | 29.99 | 0.03 | 37.44 | 0 | 0.01 | 0 | 0.25 | 0.11 | 0.01 | 0 | 0.6 | 0.15 |
| **AW10/6** | 0 | 0.03 | 0.01 | 0 | 0 | 0 | 3.92 | 1.73 | 4.41 | 27.43 | 0 | 10.77 | 0 | 7.01 | 0.02 | 0 | 1.29 | 0.13 | 2.98 | 0.02 | 0 | 38.35 | 0.33 |
| **AW11/6** | 0.02 | 0.01 | 0.03 | 0 | 0 | 0.01 | 5.33 | 4.52 | 1.73 | 56.36 | 0.01 | 19.82 | 0 | 6.61 | 0.14 | 0 | 0.01 | 0.02 | 0.6 | 0 | 0 | 0.08 | 2.39 |
| **AW3/6** | 0 | 0.4 | 0.12 | 0 | 0 | 0.02 | 3.3 | 0.11 | 18.57 | 0.56 | 0 | 10.49 | 0 | 41.01 | 0 | 0.05 | 0.01 | 0.2 | 2.3 | 0.03 | 0 | 16.21 | 4.58 |
| **AW6/6** | 0 | 0.09 | 0.04 | 0 | 0.02 | 0.02 | 4.64 | 0.53 | 10.17 | 3.89 | 0.49 | 31.02 | 5.21 | 34.96 | 1.88 | 0 | 0 | 0.06 | 0.4 | 0 | 0 | 0.13 | 1.73 |
| **AW7/6** | 0 | 0.03 | 0.4 | 0 | 0.05 | 0.01 | 4.32 | 3.03 | 19.12 | 16.74 | 0 | 44.43 | 0 | 8.68 | 0.01 | 0 | 0.01 | 0.24 | 0.25 | 0.01 | 0 | 0.09 | 0.95 |
| **AW8/6** | 0 | 0.02 | 0.03 | 0 | 0.01 | 0.05 | 6.72 | 0.63 | 1.82 | 18.69 | 0.24 | 4.5 | 0.13 | 33.95 | 0.17 | 0.01 | 0.04 | 30.04 | 0.06 | 0.01 | 0.01 | 0.23 | 0.06 |
| **AW9/6** | 0 | 0 | 0 | 0 | 0 | 0.02 | 2.7 | 0.63 | 0.38 | 10.63 | 0.01 | 12.18 | 0.08 | 12.33 | 0.14 | 0 | 0 | 0.05 | 3 | 0.01 | 0 | 55.74 | 0.04 |
| **AW3/12** | 0 | 0.35 | 0.06 | 0 | 0 | 0.01 | 2.9 | 0.19 | 1.22 | 7.79 | 0.03 | 3.96 | 0 | 67.69 | 0 | 0.04 | 0.01 | 0.1 | 1.56 | 0.01 | 0 | 9.76 | 2.05 |
| **AW4/12** | 0.01 | 0.19 | 0.12 | 0 | 0 | 0.09 | 4.28 | 0.11 | 9.69 | 1.3 | 0.31 | 5.41 | 11.27 | 52.25 | 0 | 0.04 | 0.01 | 0.12 | 1.94 | 0 | 0.01 | 0.97 | 9.76 |
| **AW5/12** | 2.02 | 0.11 | 0.48 | 2.21 | 0 | 0.03 | 4.02 | 0.16 | 2.19 | 0.78 | 0.03 | 24.92 | 0.16 | 51.21 | 0.18 | 0.56 | 0.02 | 0.21 | 0.95 | 0.22 | 0 | 0.12 | 4.83 |
| **AW5/24** | 0.02 | 0.19 | 0.8 | 0.01 | 0 | 6.76 | 1.85 | 0.01 | 0.53 | 0.06 | 3.66 | 7.91 | 0.05 | 67.02 | 0 | 0.19 | 0.17 | 0.65 | 0.42 | 0.04 | 0 | 0.15 | 2.76 |
| **BW1/0** | 0 | 0.01 | 0.01 | 0 | 0 | 0 | 3.41 | 0.36 | 8.36 | 0.37 | 0 | 67.93 | 0 | 0.01 | 0 | 0 | 0 | 0.01 | 2.11 | 0 | 0 | 0.07 | 15.84 |
| **BW2/1** | 0 | 0.01 | 0.14 | 0.02 | 0 | 0.01 | 3.79 | 4.63 | 3.57 | 17.99 | 0 | 64.71 | 0 | 0.57 | 0 | 0 | 0 | 0.01 | 0.74 | 0 | 0 | 0.24 | 3.06 |
| **BW1/6** | 0 | 0.2 | 0.08 | 0 | 0 | 0.02 | 4.83 | 3.98 | 0.8 | 53.67 | 0.05 | 20.41 | 0 | 7.26 | 0 | 0 | 0 | 0.05 | 1.24 | 0.13 | 0 | 0.07 | 5.66 |
| **BW3/6** | 0.05 | 0.44 | 0.11 | 0.05 | 0 | 0.39 | 5.29 | 2.45 | 3.48 | 18.95 | 0.02 | 37.62 | 0.03 | 27.01 | 0 | 0 | 0 | 0.06 | 0.13 | 0.03 | 0 | 0.7 | 0.09 |
| **BW3/12** | 0.08 | 1.4 | 0.17 | 0.06 | 0 | 0.33 | 3.5 | 0.34 | 0.75 | 8.46 | 0.03 | 9.49 | 0.01 | 67.44 | 0 | 0.03 | 0 | 0.09 | 0.3 | 0.09 | 0.01 | 1.96 | 0.08 |
| **BW4/12** | 0.14 | 0.01 | 0.68 | 0 | 0 | 0.08 | 5.25 | 0.47 | 0.79 | 24.15 | 0.02 | 4.22 | 0.02 | 41.6 | 2.57 | 0.04 | 0 | 0.16 | 2.16 | 0.05 | 0 | 0.73 | 11.84 |
| **CW1/0** | 0 | 0 | 0.04 | 0 | 0 | 0 | 0.88 | 0.69 | 23.01 | 0.44 | 0 | 72.6 | 0 | 0.08 | 0 | 0 | 0.01 | 0.01 | 0.27 | 0 | 0 | 0.01 | 1.23 |
| **CW2/1** | 0 | 0 | 0.01 | 0 | 0 | 0.01 | 1.33 | 1.4 | 16.06 | 2.48 | 0 | 76.88 | 0 | 0.39 | 0 | 0 | 0 | 0 | 0.21 | 0 | 0 | 0 | 1.04 |
| **CW2/2** | 0 | 0 | 0.02 | 0 | 0 | 0 | 1.81 | 1.49 | 22.01 | 2.94 | 0 | 65.98 | 0.14 | 2.79 | 0 | 0 | 0 | 0 | 0.32 | 0 | 0 | 0 | 2 |
| **CW1/6** | 0 | 0.04 | 0.16 | 0 | 0.01 | 0.23 | 3.57 | 1.91 | 4.21 | 12.35 | 0.01 | 53.82 | 0.06 | 19.76 | 0 | 0.03 | 0 | 0.01 | 0.17 | 0.03 | 0 | 0.01 | 1.03 |
| **CW3/6** | 0 | 0.02 | 0.03 | 0 | 0.01 | 0.1 | 4.61 | 1.55 | 21.64 | 7.53 | 0.01 | 36.43 | 0.16 | 21.4 | 0.03 | 0.01 | 0 | 0.03 | 0.51 | 0.01 | 0 | 0 | 3 |
| **CW2/7** | 0 | 0.04 | 0.15 | 0 | 0 | 0.01 | 4.07 | 1.07 | 15.3 | 5.28 | 0 | 39.04 | 0.76 | 24.97 | 0 | 0 | 0 | 0.02 | 0.92 | 0.01 | 0 | 0 | 5.35 |
| **CW2/9** | 0 | 0.03 | 0.33 | 0 | 0 | 0.01 | 4.23 | 0.59 | 24.45 | 2.75 | 0 | 22.07 | 0.02 | 32.98 | 0 | 0 | 0 | 0 | 1.17 | 0.01 | 0 | 0 | 9.1 |
| **CW2/12** | 0 | 0.03 | 0.24 | 0 | 0 | 0 | 3.76 | 0.36 | 30.8 | 1.68 | 0 | 18.04 | 0.23 | 30.44 | 0 | 0 | 0 | 0.01 | 1.33 | 0 | 0 | 0 | 10.42 |
| **CW3/12** | 0 | 0.04 | 0.1 | 0 | 0.01 | 0.13 | 3.18 | 0.3 | 44.36 | 1.15 | 0 | 10.3 | 0.27 | 32.64 | 0.37 | 0.02 | 0 | 0.02 | 0.34 | 0.01 | 0 | 0.02 | 2.44 |
| **CW4/12** | 0 | 0.05 | 0.04 | 0 | 0 | 0.04 | 2.71 | 0.15 | 1.27 | 3.36 | 0 | 9.41 | 0.03 | 76.03 | 0 | 0 | 0 | 0.02 | 0.46 | 0.06 | 0 | 0.34 | 2.02 |
| **DW1/0** | 0 | 0 | 0 | 0 | 0 | 0.08 | 1.92 | 0.72 | 57.2 | 0.1 | 0 | 22.71 | 0 | 0.05 | 0 | 0.01 | 0 | 0.01 | 1.87 | 0.02 | 0 | 0.04 | 13.61 |
| **DW2/1** | 0 | 0 | 0 | 0 | 0.05 | 0.03 | 1.04 | 0.45 | 12.48 | 0.28 | 0 | 82.35 | 0.01 | 0.08 | 0 | 0 | 0 | 0.01 | 0.56 | 0.01 | 0 | 0.01 | 2.26 |
| **DW1/6** | 0.01 | 0.02 | 0.02 | 0 | 0 | 0.51 | 5.33 | 0.35 | 2.49 | 0.99 | 0 | 31.39 | 0 | 27.43 | 0 | 0.01 | 0 | 0.03 | 3.27 | 0.13 | 0.01 | 0.14 | 26.02 |
| **DW10/6** | 0 | 0 | 0.03 | 0 | 0 | 0.04 | 5.14 | 1.34 | 14.94 | 5.22 | 0 | 31.81 | 0 | 13.53 | 0.55 | 0 | 0 | 0 | 2.97 | 0 | 0 | 0.01 | 22.69 |
| **DW11/6** | 0 | 0.01 | 0.02 | 0 | 0.02 | 0.06 | 4.63 | 2.5 | 14.74 | 16.24 | 0.02 | 35.37 | 0 | 14.12 | 1.35 | 0 | 0.06 | 0 | 1.1 | 0.01 | 0 | 0.4 | 6.02 |
| **DW3/6** | 0.01 | 0.04 | 0.06 | 0 | 0.02 | 0.31 | 5.79 | 3.5 | 8.35 | 24.43 | 0 | 30.42 | 0 | 6.44 | 0 | 0.02 | 0.01 | 0.03 | 2.44 | 0.12 | 0.06 | 2.01 | 14.26 |
| **DW6/6** | 0 | 0 | 0 | 0.01 | 0 | 0 | 3.22 | 0.3 | 7.32 | 0.13 | 0 | 73.04 | 0 | 4.72 | 0 | 0 | 0 | 0.01 | 1.55 | 0 | 0 | 0.01 | 8.44 |
| **DW7/6** | 2.1 | 0.01 | 0 | 4.75 | 0 | 0 | 3.35 | 0.62 | 21.38 | 1.25 | 0 | 33.97 | 0 | 20.02 | 0.01 | 0 | 0 | 0.02 | 0.85 | 0.01 | 0 | 0.01 | 4.18 |
| **DW8/6** | 0.01 | 0.02 | 0.02 | 0 | 0 | 0.03 | 2.59 | 0.6 | 10.36 | 1.34 | 0 | 67.87 | 0 | 9.16 | 0 | 0 | 0 | 0.02 | 0.68 | 0 | 0 | 0.8 | 3.5 |
| **DW9/6** | 0 | 0.02 | 0.02 | 0 | 0 | 0.03 | 3.54 | 0.51 | 12.57 | 0.33 | 0.02 | 58.47 | 0.05 | 16 | 0 | 0.02 | 0 | 0.01 | 1.12 | 0.03 | 0.03 | 0.04 | 5.3 |
| **DW3/12** | 0.01 | 0.12 | 0.07 | 0 | 0 | 0.56 | 5.5 | 1.11 | 8.65 | 13.06 | 0.07 | 13.44 | 0 | 30.85 | 0 | 0.04 | 0.02 | 0.06 | 2.67 | 0.26 | 0.12 | 5.5 | 13.42 |
| **DW4/12** | 0 | 0.03 | 0.27 | 0 | 0.27 | 3.79 | 5.94 | 0.67 | 1.3 | 34.55 | 0.24 | 2.77 | 0.04 | 27.56 | 0.5 | 0.15 | 1.34 | 0.4 | 2.02 | 0.47 | 0.01 | 11.87 | 0.02 |
| **DW5/24** | 0 | 0.13 | 0.25 | 0.02 | 0.12 | 0.47 | 1.87 | 0.12 | 2.01 | 1.85 | 0.04 | 9.06 | 0 | 61.93 | 0.11 | 0.46 | 2.35 | 0.5 | 0.77 | 9.63 | 1.59 | 0.55 | 0.02 |
| **EW1/0** | 0 | 0 | 0.13 | 0 | 0.85 | 0.02 | 1.89 | 0.74 | 31.31 | 0.42 | 0 | 53.87 | 0 | 0.04 | 0 | 0 | 0 | 0.02 | 1.3 | 0.01 | 0 | 0.03 | 7.61 |
| **EW2/1** | 0 | 0 | 0.08 | 0 | 0.02 | 0.07 | 2.31 | 3.21 | 10.51 | 9.49 | 0 | 72.04 | 0.02 | 0.37 | 0 | 0.01 | 0 | 0.02 | 0.29 | 0 | 0 | 0.14 | 0.98 |
| **EW2/2** | 0 | 0 | 0.08 | 0 | 0.01 | 0.04 | 2.89 | 3.43 | 12.93 | 9.93 | 0 | 66.39 | 0.02 | 1.03 | 0 | 0.01 | 0 | 0.01 | 0.46 | 0.02 | 0 | 0.16 | 2.06 |
| **EW10/6** | 0 | 0.03 | 0.2 | 0 | 0.01 | 0.25 | 5.21 | 2.64 | 11.18 | 24.66 | 0 | 30.44 | 1.07 | 21.98 | 0.01 | 0.02 | 0.01 | 0.11 | 0.09 | 0.24 | 0 | 0.35 | 0.03 |
| **EW11/6** | 0 | 0.01 | 0.11 | 0 | 0.02 | 0.09 | 6.27 | 3.35 | 2.73 | 34.61 | 0.01 | 26.99 | 0.23 | 19.14 | 0 | 0.06 | 0.07 | 0.04 | 0.62 | 0.35 | 0 | 0.22 | 2.6 |
| **EW3/6** | 0 | 0.01 | 0.2 | 0 | 0 | 0.36 | 6.85 | 2.84 | 7.8 | 20.87 | 0.01 | 24.08 | 0.1 | 17.44 | 0.02 | 1.41 | 0.07 | 6.37 | 1.43 | 0.18 | 0 | 2.14 | 4.97 |
| **EW6/6** | 0 | 0.04 | 0.09 | 0 | 1.05 | 0.02 | 5.75 | 5.39 | 4.94 | 23.06 | 0.06 | 51.54 | 0.03 | 5.26 | 0 | 0.01 | 0.02 | 0.01 | 0.23 | 0.01 | 0 | 0.07 | 0.86 |
| **EW7/6** | 0.01 | 0.01 | 0.03 | 0 | 0 | 0.04 | 4.51 | 4.08 | 8.2 | 16.27 | 0 | 55.41 | 0.46 | 6.37 | 0.01 | 0.02 | 0 | 0.01 | 0.64 | 0.06 | 0 | 0.36 | 2.52 |
| **EW8/6** | 0 | 0.1 | 0.28 | 0 | 0.06 | 0.49 | 4.84 | 1.96 | 2.11 | 8.56 | 0.12 | 50.28 | 0.81 | 18.81 | 0 | 0.14 | 0.06 | 0.13 | 0.89 | 0.26 | 0 | 1.14 | 3.34 |
| **EW9/6** | 0 | 0.01 | 0.14 | 0 | 0 | 0.24 | 4.77 | 4.67 | 20.44 | 34.67 | 0 | 28.2 | 0.18 | 3.77 | 0 | 0.08 | 0 | 0.06 | 0.2 | 0.01 | 0 | 0.29 | 0.82 |
| **EW2/7** | 0 | 0.04 | 0.56 | 0 | 1.64 | 0.11 | 5 | 4.66 | 3.67 | 25.17 | 0 | 47.1 | 0.32 | 8.61 | 0 | 0.03 | 0.01 | 0.03 | 0.15 | 0.15 | 0 | 0.18 | 0.45 |
| **EW2/9** | 0.16 | 0.04 | 0.47 | 0 | 0.01 | 0.17 | 6.38 | 2.37 | 4.6 | 42.78 | 0.01 | 10.22 | 1.22 | 23.22 | 0.02 | 0.06 | 0.04 | 0.03 | 0.69 | 0.26 | 0 | 2.16 | 1.03 |
| **EW2/12** | 0.26 | 0.13 | 0.52 | 0 | 0.02 | 0.41 | 5.76 | 0.74 | 2.26 | 32.36 | 0.01 | 5.04 | 0.42 | 39.28 | 0 | 0.09 | 0.07 | 0.09 | 1.23 | 0.66 | 0 | 3.37 | 4.09 |
| **EW3/12** | 0 | 0.09 | 0.35 | 0 | 0 | 0.3 | 3.65 | 1.18 | 11.81 | 8.04 | 0 | 31.58 | 0 | 8.51 | 0 | 2.17 | 0.16 | 8.54 | 1.52 | 0.64 | 0 | 5.51 | 11.01 |
| **FW1/0** | 0 | 0 | 0.06 | 0 | 0.01 | 0.13 | 1.4 | 0.71 | 30.45 | 0.08 | 0 | 59.72 | 0 | 0.01 | 0 | 0 | 0 | 0.05 | 0.75 | 0.01 | 0 | 0.03 | 4.63 |
| **FW2/1** | 0 | 0.01 | 0.18 | 0 | 0.01 | 0.03 | 1.59 | 1.09 | 28.25 | 1.56 | 0.01 | 62.01 | 0.01 | 0.49 | 0 | 0 | 0 | 0.53 | 0.73 | 0.01 | 0 | 0.02 | 2.97 |
| **FW2/2** | 0 | 0.02 | 0.47 | 0 | 0.01 | 0.13 | 2.5 | 1.22 | 33.68 | 2.28 | 0.02 | 49.19 | 0.23 | 3.51 | 0 | 0.04 | 0.02 | 0.97 | 0.65 | 0.11 | 0 | 0.03 | 3.96 |
| **FW1/6** | 0 | 0.07 | 0.52 | 0 | 0.03 | 0.43 | 4.46 | 1.9 | 4.92 | 11.22 | 0.42 | 42.66 | 0.02 | 17.47 | 0 | 0.08 | 0.07 | 0.57 | 1.08 | 1.84 | 0.01 | 0.71 | 5.14 |
| **FW3/6** | 0 | 0.55 | 0.23 | 0 | 0.01 | 0.28 | 3.06 | 0.12 | 20 | 0.07 | 0 | 12.36 | 0.01 | 47.53 | 0 | 0.03 | 0.07 | 0.6 | 1.24 | 0.35 | 0.16 | 7.62 | 3.39 |
| **FW2/7** | 0 | 0.07 | 1.26 | 0 | 0 | 0.26 | 6.47 | 2.01 | 12.62 | 13.61 | 0.45 | 31.05 | 0.52 | 18.44 | 0 | 0.16 | 0.08 | 4.2 | 0.92 | 0.61 | 0 | 0.24 | 4.17 |
| **FW2/9** | 0 | 0.06 | 1.55 | 0 | 0 | 0.21 | 6.78 | 1.47 | 7.64 | 19.62 | 0.46 | 14.34 | 0.69 | 29.51 | 0 | 0.2 | 0.18 | 6.53 | 1.08 | 1.38 | 0 | 0.31 | 4.55 |
| **FW2/12** | 0 | 0.08 | 2.59 | 0 | 0.02 | 0.35 | 6.08 | 0.44 | 3.2 | 10.61 | 2.46 | 7.52 | 1.09 | 41.61 | 0 | 0.42 | 0.16 | 8.51 | 1.45 | 1.44 | 0 | 0.47 | 7.75 |
| **FW3/12** | 0 | 1.59 | 0.36 | 0 | 0.01 | 0.17 | 2.6 | 0.16 | 15.79 | 0.03 | 0 | 18.23 | 0 | 29.16 | 0 | 0.05 | 0.15 | 1 | 1.62 | 0.63 | 0.26 | 16.48 | 4.98 |
| **FW4/12** | 0 | 0.15 | 0.67 | 0 | 0 | 0.08 | 2.47 | 0.22 | 9.58 | 6.82 | 0 | 4.14 | 0 | 51.51 | 0 | 0.03 | 0.04 | 0.46 | 1.33 | 0.28 | 1.02 | 10.41 | 3.16 |
