# Supplementary material for: The Interrelationship Between Microbiota and Peptides During Ripening as a Driver for Parmigiano Reggiano Cheese Quality
Source: Front Microbiol. 2020 Oct 2;11:581658. doi: 10.3389/fmicb.2020.581658 (PMC7561718; doi:10.3389/fmicb.2020.581658)
Supplement: Supplementary file 3 [file Table_3.DOCX]

**Supplementary Figure 1.** Diversity indices at different ripening times as determined by HTS. Simpson’s (S) diversity index is represented from solid orange line, Evennes (E) by a gray solid line, and Shannon Entropy (H) by a blue solid line.
