# Supplementary material for: The Interrelationship Between Microbiota and Peptides During Ripening as a Driver for Parmigiano Reggiano Cheese Quality
Source: Front Microbiol. 2020 Oct 2;11:581658. doi: 10.3389/fmicb.2020.581658 (PMC7561718; doi:10.3389/fmicb.2020.581658)
Supplement: Supplementary file 4 [file Table_4.DOCX]

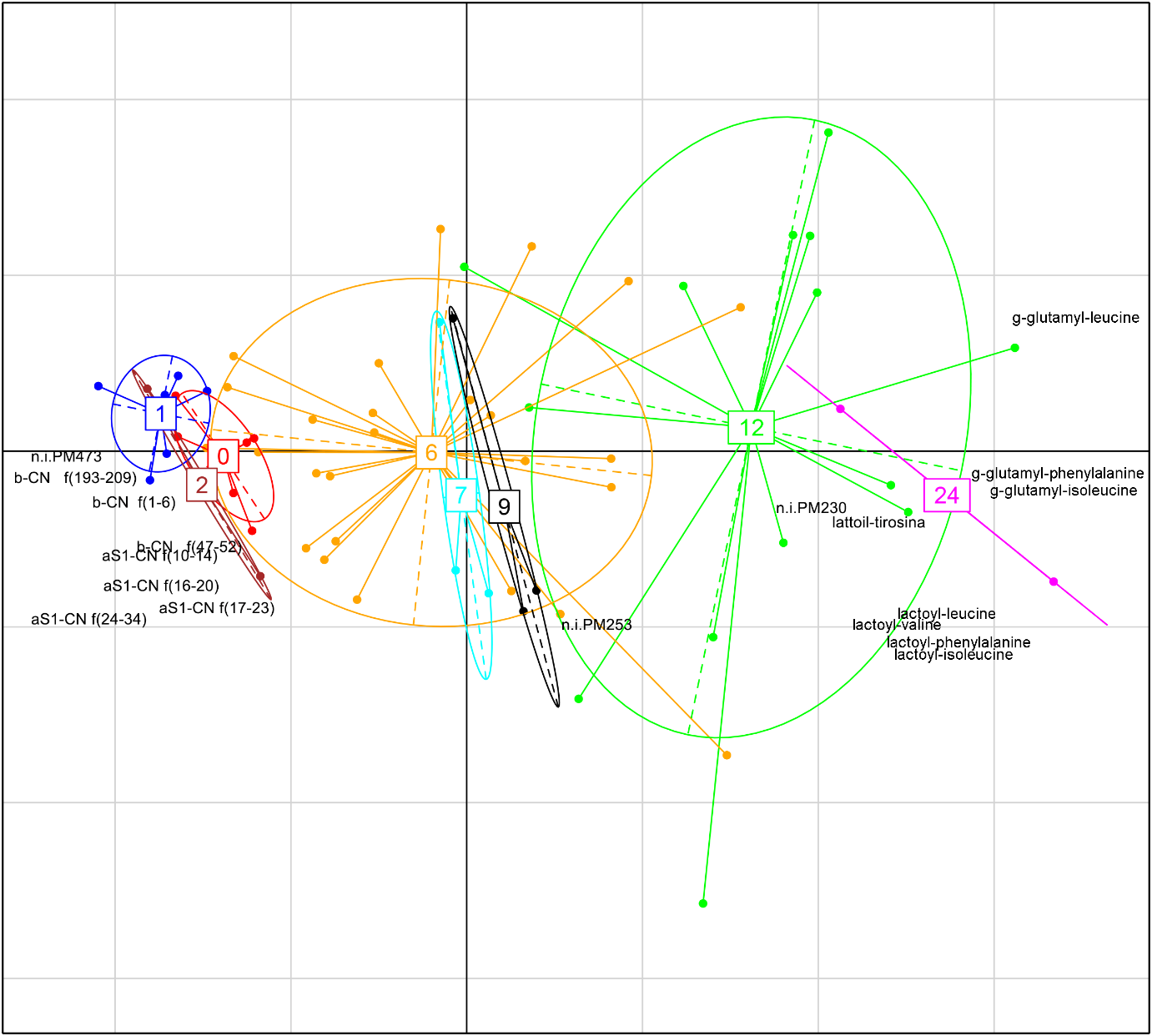


**Supplementary Figure 2**. Clustering of cheese samples according to their microbial composition, superimposed with data regarding the measured peptide fraction. A ripening trend exists among the variables: samples are clustered according to aging time: 0) 48 h curds; 1) 1 mo. old cheeses; 2) 2 mo. old cheeses; 6) 6 mo. old cheeses; 7) 7 mo. old cheeses; 7) 9 mo. old cheeses; 12) 12 mo. old cheeses; 24) 24 mo. old cheeses.
